# The coordinating evaluation and spatial correlation analysis of CSGC: A case study of Henan province, China

**DOI:** 10.1371/journal.pone.0174543

**Published:** 2017-06-08

**Authors:** Mingxia Xie, Jiayao Wang, Ke Chen

**Affiliations:** 1 Changjiang Spatial Information Technology Engineering CO.LTD, Wuhan, China; 2 Institute of Surveying and Mapping, Information Engineering University, Zhengzhou, China; University of Rijeka, CROATIA

## Abstract

This study investigates the basic characteristics and proposes a concept for the complex system of geographical conditions (CSGC). By analyzing the DPSIR model and its correlation with the index system, we selected indexes for geographical conditions according to the resources, ecology, environment, economy and society parameters to build a system. This system consists of four hierarchies: index, classification, element and target levels. We evaluated the elements or indexes of the complex system using the TOPSIS method and a general model coordinating multiple complex systems. On this basis, the coordination analysis experiment of geographical conditions is applied to cities in the Henan province in China. The following conclusions were reached: ①According to the pressure, state and impact of geographical conditions, relatively consistent measures are taken around the city, but with conflicting results. ②The coordination degree of geographical conditions is small among regions showing large differences in classification index value. The degree of coordination of such regions is prone to extreme values; however, the smaller the difference the larger the coordination degree. ③The coordinated development of geographical conditions in the Henan province is at the stage of the point axis.

## Introduction

### The complex system of geographical conditions (CSGC)

Geographical conditions mainly refer to the spatial distribution, characteristics and interrelations of natural and human geographical elements. It constitutes an essential part of the basic national conditions [1]. In the narrow sense, it refers to the basic conditions and characteristics of the natural environment and its resources which are closely connected within a geographical area. In a broad sense, it refers to the association of various national conditions (including natural environments and natural resources, scientific and technological education status, economic development status, political status, social status, cultural traditions, international environments, international relations, etc.) to certain attributes in a geographical area. This is used to reveal the spatial-temporal evolution and internal relationships of economic and social development [1].

Currently, institutions and researchers studying statistical analysis of geographical conditions in China, consider the method as a predominantly engineering project. Instead of carrying out preliminary theoretical analyses and discussions on geographical conditions, these statistical analyses commonly refer to basic surveying and mapping, traditional cartography, spatial analysis, GIS system development and other relevant ideas and models. The approach to solving problems in complex systems with engineering techniques is usually not applied to the complex, multi-dimensional subject of geographical conditions which has a varied theoretical and technical background. Theoretical research is lagging behind applied research causing difficulties in their integration.

Geographical conditions include resources, ecology, environment, economy, and society. These systems are different in nature, structure, function and development. However, their existence and development are constrained by the structures and functions of other systems. There are extensive nonlinear interactive mechanisms within the various elements, and mutual connections and constraints have demonstrated their complexity and comprehensiveness which are essential attributes of geographical conditions. Current research is conducted on binary [2–5], ternary [6–11], and quaternary systems [12,13], such as: “resources-environment”, “environment-economy”, “resources-environment-economy”, “resources-environment-ecology”, “resources-environment-economy-society”. We have found the resources, ecology, environment, economy, society and other relevant elements cannot be isolated when studying geographical conditions. To undertake a more systematic analysis, these elements must be treated as an entity., Geographical conditions also have multi-dimensional structural, non-linear, sequential and coordinate symbiosis characteristics to accompany the integrity, complexity and comprehensiveness. Therefore, geographical conditions and the complex system have consistent basic characteristics. The degree of coordination is an important method to study the complex system and achieve global coordination and optimal design. It will enable coordination and support in various subsystems to achieve the general tasks and goals of the system consistent with the geographical conditions statistical analysis. Geographical conditions form a complex system that integrates resources, environment, ecology, economy and society that can be quantitatively described as:

$$\begin{array}{l} GCCS\subseteq \left\{S_{1},S_{2},S_{3},S_{4},S_{5},R_{a},T\right\}\\ S_{i}\subseteq \left\{I_{i},C_{i},F_{i}\right\},i=1,2,\cdots ,5 \end{array}$$ (1)

where *S*_1_, *S*_2_, *S*_3_, *S*_4_, *S*_5_ represent various subsystems constituting the CSGC; *R*_*a*_ is the correlation system, a set of correlations which includes correlations among various subsystems and among the indexes of these subsystems. These correlations include multiple correlations of the underlying structures of the subsystems, and the complex multi-directional relations formed by related factors [14–16]. They possess basic attributes such as diversity [17], interactivity, hierarchy and dynamics [18,19]. *T* represents the time sequence of CSGC. *I*_*i*_, *i* = 1,2,⋯,5 represents the indexes of the subsystems (dynamic sequential parameters in the complex system); *C*_*i*_, *i* = 1,2,⋯,5 indicates the structures of the subsystems; and *F*_*i*_, *i* = 1,2,⋯,5 shows the calculation functions, or methods, of the indexes forming the subsystems. The specific goals of geographical conditions quantitatively express *R*_*a*_ (the relations in the complex “resources-environment-ecology-economy-society” system). However, methods of measuring these relations have not been fully investigated. The complex system theory has provided an important method and the coordination degree has offered a new method to measure the relations among elements of geographical conditions.

### The coordinating evaluation model

Coordinating a complex system reflects the relations between various subsystems and among their internal indexes. The coordination degree measures the quality of harmony and consistency of a system, or of its internal elements during the developmental process. It reflects the transition from a disorderly to an orderly state defined by a quantitative coordinative index [20–23]. The coordination degree evaluation model can be divided into three types: distance, change and integrated types (Table 1). The distance type constitutes one of the mainstream methods of coordination degree evaluation focusing on a long-term macroeconomic point of view. It is a relatively static model using a certain distance between the systems as a benchmark to measure the degree of coordination. The change type is a dynamic evaluation model which measures the relative degree of change between systems and uses the consistency of dynamic changes between each subsystem from a transient local point of view. The integrated type does not consider the independence of movement and the intrinsic link between systems and various elements within the system from a holistic perspective. Instead, it considers the system as an organic entity and examines its state of development by assuming the organic system conserves its energy [7].

**Table 1:** The comparison between different types of coordination degree evaluation models.

category	feature	common method
distance type	long-term, macroeconomic, static, whole, complex	deviation coefficient method, membership function method, gene coefficient method, set pair analysis, data envelopment analysis and so on;
change type	transient, dynamic, local, complex	grey system model, differential coefficient method and so on;
integrated type	long-term, static, whole, simple	geometric mean method, coupling degree method and so on

Most existing coordination degree models are suitable for coordination analysis of a binary complex system. However, fewer studies [10–13] proposed the usage of coordination analysis of a multiple complex system and commonly encountered some problems:

1. The analysis is only suitable for a specific research and is non-universal;
2. The calculation process is complex and it is difficult to explain the meaning of the mathematical expressions.

To solve these problems, we need to evaluate the state of development of the subsystem or index using a technique for order preference by similarity to an ideal solution (TOPSIS), for which the general evaluation model for coordination degree of multiple complex systems is designed.

## Establishing data sources and index systems for the coordinating analysis

### Study area and data sources

Our area of study is the Henan province which is located in the eastern part of China in the middle and lower reaches of the Yellow River. To the east, the region is close to Anhui and Hubei, to Hebei and Shanxi to the north, to Shaanxi to the west and Hubei to the south. The region extends across the Haihe, Yellow, Huaihe and Yangtze rivers which are four major river basins, linking the eastern and western areas. The Henan province is also the most populous province of China, the first in agriculture and an emerging industrial and labor-exporting province. Arable land forms 71.792 million ha, and mountainous areas 74,000 km^2^ totaling 44.3% of the province area. Plains and basins cover an area of 93,000 km^2^, nearly 55.7% of the total area. The Henan province is divided into 18 research units included in municipal administrative divisions. Data concerning the land usage is provided by the 2015 geographical census and the resources, environment, ecological, social and economic data is extracted from the Statistical Yearbook and urban planning information at the provincial and city levels [24,25].

### The index system for coordinating analysis of geographical conditions

When establishing an index system for coordinating analysis of CSGC, the emphasis should be on specific circumstances and the indexes should be selected to characterize the status of geographical conditions, while ensuring data accessibility. Indexes should first be classified, followed by an analysis of the significance of various indexes during the coordinating analysis. Finally, the index should be based upon selected indexes and their classification pattern, to reflect the spatial distribution, impact, and interactions of the geographical information. The purpose is to reveal objectively and accurately its spatial distribution rules and evolution trends relative to resources, the ecology, environment, economy and society [26].

**Fig 1:**
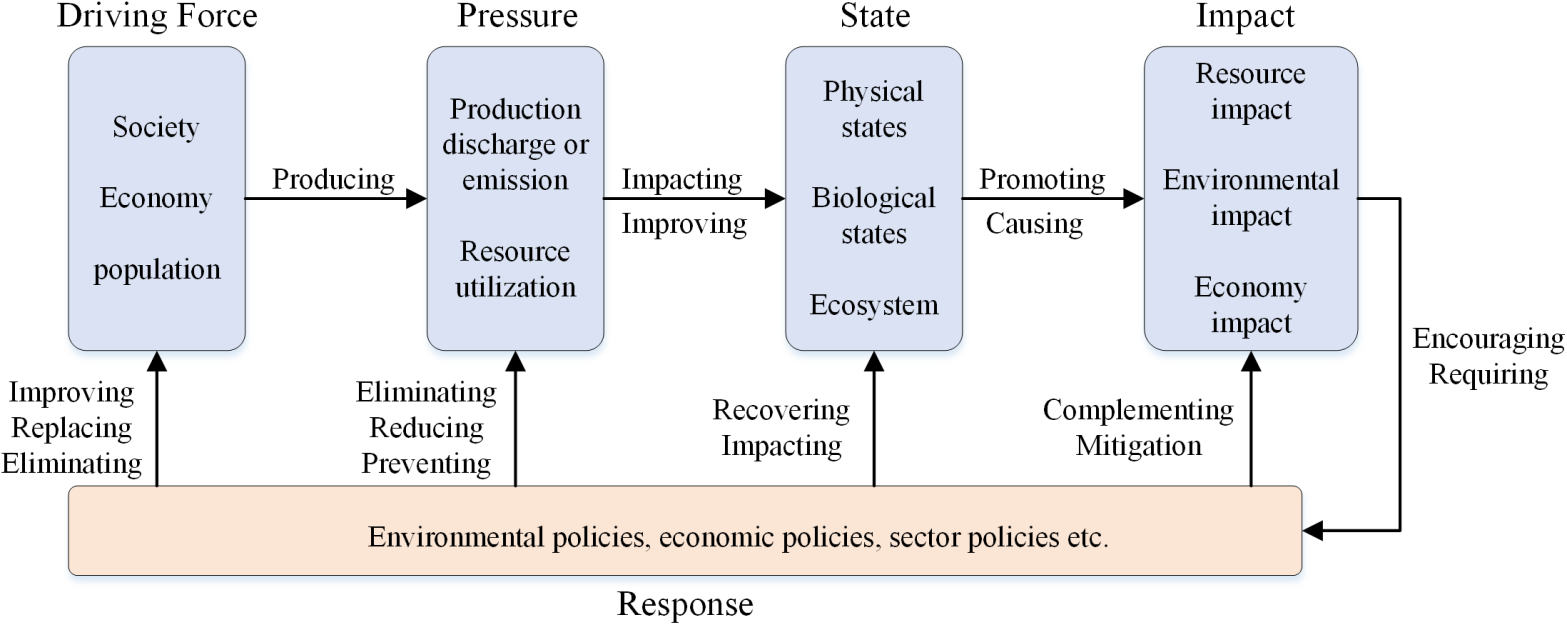
DPSIR model and its inner connections [35].

The Driving-Force-Pressure-State-Impact-Response (DPSIR) model describes a chain of cause and effect in environmental problems. Long-term driving forces, socio-economic development, and population increases impose pressure upon the environment and modify the ecological state. The impacts on the environment lead human beings to change the state of the ecology. These modifications affect a complex system composed of society, economy, and population, or directly act on environmental pressure, state, and effects. The contact between the elements is shown in Fig 1. The DPSIR conceptual model is systematic, contains explicit hierarchies and can effectively characterize the concepts and structures of complex systems. It is often used to evaluate social [27,28] and environmental [29–32] systems, and ecosystems [33,34]. To date, no study has applied the DPSIR model to evaluate geographic national conditions. The hierarchical framework describes the dependencies of system functions and can conveniently simplify a complex system. The most important aspect of the framework is its ability to describe complex systems in a similar manner to the thinking process of humans [35,36]. The DPSIR conceptual model and its index classification system fit the relevant factors during the integration of the coordination analysis of CSGC, while using the advantages of a hierarchical framework.

There is no specific approach to screen the indexes; these are mainly artificially selected according to the characteristics of the CSGC. In this paper, the index selection combines theoretical and frequency analyses. First, we searched through 72 recently published papers in journals and conferences abstracts on geographical conditions and related fields. These papers are searched using the China Journal Full-text Database of CNKI through setting the subjects such as "geographical conditions", "resources" and "evaluation index", "environment", "evaluation index" "ecology", "evaluation index", “economy", "evaluation index", "society", "evaluation index", and selected according to the journals grade. A statistical analysis was conducted based on current document requirements, followed by a frequency analysis to extract used indexes. We then performed analyses and comparisons to select factors that can best represent the development and changes in resources, ecology, environment, economy, and society after considering the background characteristics of geographical conditions in China, the connotations and features of complex systems, the main questions being asked, the index establishment techniques, etc. Finally, some redundant indexes (which have different names but give the same information) selected by frequency and theoretical analysis were excluded. Twenty two key indexes affecting the coordination analysis of CSGC were extracted (Table 2). The selected indexes were subjectively classified by experts, following the definitions of various factors within the DPSIR model.

**Table 2:** The index selection for statistical analysis of CSGC.

Subsystem	Index	Connotation
Resources	Degree of land use	Scope and depth of land use.
	Vegetation coverage index	Percentage of forest relative to the total area.
	Density index of river networks	Percentage of the total length of rivers, water surface area and amount of water resources within the evaluated area. Reflects the richness of the water in the area.
	Per capita residential area	Ratio of residential area to the total amount of the permanent residential population of the city.
	Per capita urban road area	Ratio of urban road area to the total amount of permanent residential population of the city.
Environment	Air quality index	Maximum value of air quality index related to various pollutants.
	Sewage treatment rate	Ratio of treated domestic sewage and industrial wastewater to the total discharge of sewage
	Percentage of environmental protection investment relative to the total expenditure	Percentage of funds devoted to environmental protection relative to the total expenditure
Ecology	Biological abundance index	Indirectly reflects the biological richness of the evaluated region via the variations in the number of species in different types of ecosystems per unit area.
	Ecological civilization index	Ratio of the regional ecological footprint of the GDP per capita to the national ecological footprint of GDP per capita
	Proportion of ecological land use	Percentage of ecological land use relative to the total land area.
	Percentage of ecological restoration relative to the total expenditure	Percentage of funds devoted to ecological restoration relative to the total expenditure
Economy	Urbanization rate	Ratio of urban population to the total population. An important symbol of national or regional economic development, and an essential index of the level of social organization and management in a country or region.
	GDP per capita	Ratio of GDP to the permanent resident population (or household population). An effective tool to understand the macroeconomic performance of a country or region.
	Resident consumption index	The relative price level of a group of representative consumer goods and services measured over time.
	Per capita income ratio of urban and rural residents	Income per capita of urban and rural residents.
	Engel’s coefficient	Ratio of total personal food expenditure to the total personal consumption expenditure
Society	Per capita park area	Ratio of park area space to the non-agricultural population.
	Number of community service facilities	Number of community service facilities.
	Percentage of social security expenditure relative to the total expenditure	Percentage of social security expenditure relative to the total expenditure.
	Proportion of people with medical insurance	Percentage of people participating in medical insurance relative to the total population.
	Proportion of pension insurance	Percentage of people having pension insurance relative to the total population.

We assume there is a system of classified indexes than can reflect the nature of the evaluated objects. Therefore, if the adopted system of classified indexes is similar to the one assumed, then the data obtained from evaluating with the index system will better reflect the nature of the evaluated and analyzed objects. The index system will also be more stable and reliable.

**Definition 1.** *The optimal index classification*. *The classification of the indexes related to geographic national conditions by experts using the DPSIR model is C =* {*c*_1_, *c*_2_, ⋯, *c*_*n*_}, *where n = the number of experts*, *c*_*i*_ *=* {*c*_*i*1_, *c*_*i*2_, *c*_*i*3_, *c*_*i*4_, *c*_*i*5_}, *i* = 1,2,⋯,*n*, *and 1*, *2*, *3*, *4 and 5 represent five factors*, *i*.*e*. *D*, *P*, *S*, *I and R*, *in the DPSIR model*, *respectively*. *An index classification c is optimal*, *when minimum of the Eq* *2* *is attained*.

$$\sum_{i=1}^{n}c_{i}-c$$ (2)

where *c*_*i*_ − *c* is the difference in classification *c*_*i*_ and *c*; it is equal to 0 for the same items in *c*_*i*_ and *c*, while it is equal to 1 for different items during the calculation. Assuming that *c*_1_ and *c*_2_ show the values presented in Table 3, the indexes in D classification between *c*_1_ and *c*_2_ are similar, the number of different indexes in the P classification between *c*_1_ and *c*_2_ is 2, the number of different indexes in the S classification is 1, the indexes in the I classification between *c*_1_ and *c*_2_ are similar, and the number of different indexes in the R classification is 3, so *c*_1_ − *c*_2_ = 2 + 1 + 3 = 6.

**Table 3:** Example of a difference calculation for the index classification.

Classification	*c*_1_	*c*_2_	Difference
D	• GDP per capita • Resident consumption index • Engel’s coefficient • Number of community service facilities	• GDP per capita • Resident consumption index • Engel’s coefficient • Number of community service facilities	0
P	• Degree of land use • Proportion of ecological land use • Vegetation coverage index • Per capita income ratio of urban and rural residents	• Proportion of ecological land use • Vegetation coverage index • Per capita income ratio of urban and rural residents • Urbanization rate	2
S	• Ecological civilization index • Biological abundance index • Proportion of people with medical insurance • Proportion of people with pension insurance	• Ecological civilization index • Biological abundance index • Proportion of people with medical insurance • Proportion of people with pension insurance • Sewage treatment rate	1
I	• Per capita residential area • Per capita urban road area • Per capita park area • Density index of river networks • Air quality index	• Per capita residential area • Per capita urban road area • Per capita park area • Density index of river networks • Air quality index	0
R	• Urbanization rate • Percentage of social security expenditure relative to the total expenditure • Percentage of ecological restoration relative to the total expenditure • Percentage of environmental protection investment relative to the total expenditure • Sewage treatment rate	• Land use degree • Percentage of social security expenditure relative to the total expenditure • Percentage of ecological restoration relative to the total expenditure • Percentage of environmental protection investment relative to the total expenditure	3

**Fig 2:**
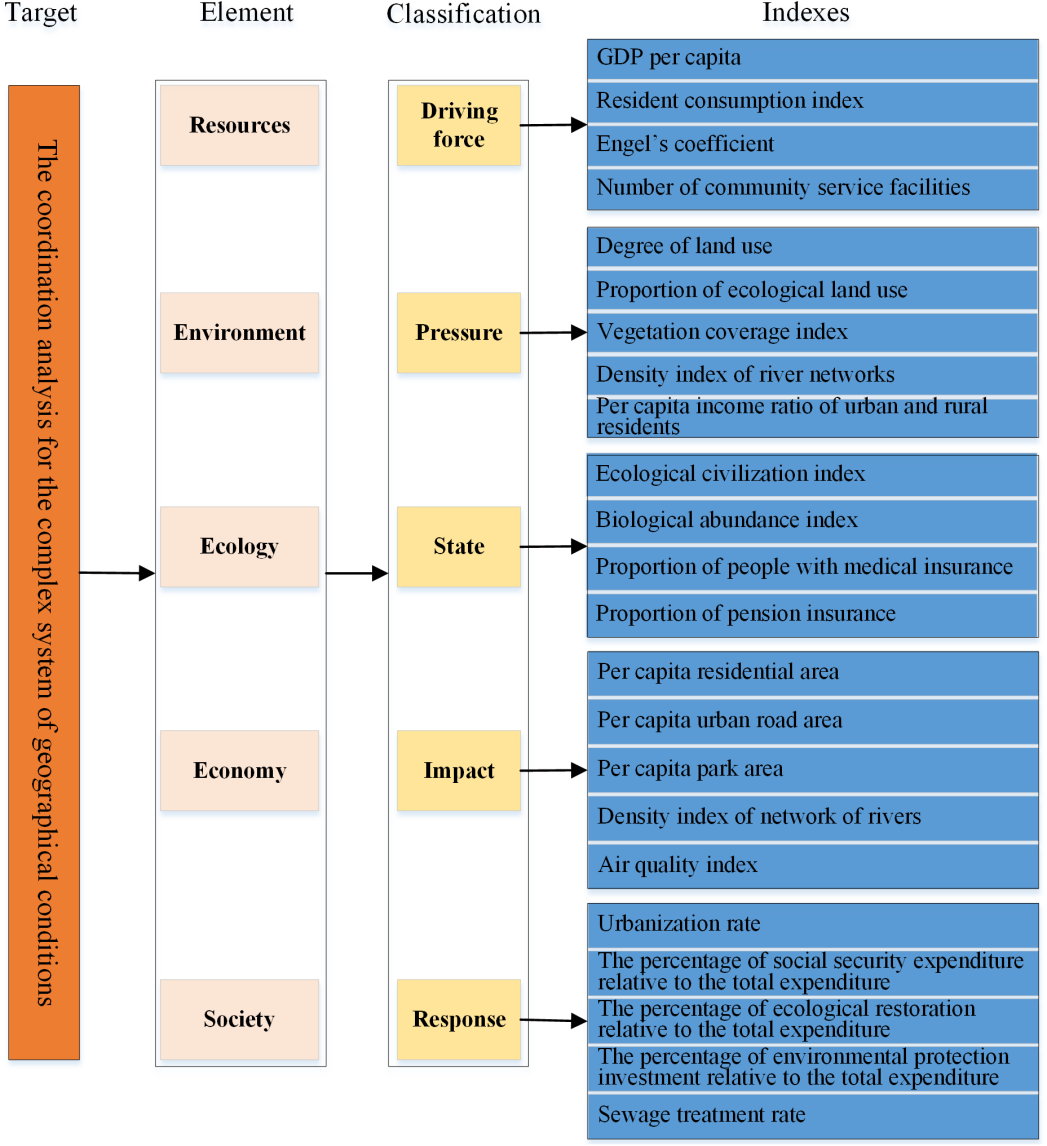
Index system for coordinating analysis of geographical conditions based on the DPSIR model.

We have chosen 30 college professors working in the fields of geoscience, environment, ecology, economy and society. We sent the statistical tables relative to all indexes and classification to the professors. The results (30 statistics tables of the survey) are collected and analyzed and we obtained the optimal evaluation system for geographic national conditions. We built an index system for the coordination analysis of CSGC (Fig 2) consisting of four hierarchies. The first is the target hierarchy which consists of an integrated coordination analysis of geographical conditions. The second hierarchy is the element hierarchy, that includes five factors such as resource, environment, ecology, economy and social. The third hierarchy forms the classification hierarchy and contains five factors influencing the evaluation and analysis of the coordination of CSGC such as the driving force, pressure, state, impact and response. The index hierarchy is the last. The core is the intermediate hierarchy that includes the element hierarchy and classification hierarchy.

## The methods of coordinating analysis

### TOPSIS

The TOPSIS method was first proposed by C.L.Hwang and K.Yoon in 1981, and categorized according to the closeness between a finite number of evaluation objects and an ideal target [37]. TOPSIS is a sorting method approximating an ideal solution and requiring a steadily increasing or decreasing utility function. “Ideal solution” and “negative ideal solution” are the two basic concepts of the TOPSIS method. The ideal solution envisages every attribute value being the best of each alternative. The negative ideal solution considers every attribute value being the worst of each alternative. The basic principle of TOPSIS method is given by the distance from evaluated objects to the ideal and negative ideal solutions. The best scenario occurs when the evaluation objects are close to the ideal solution and distant from the negative ideal solution. Otherwise, it is not optimal.

### The general evaluation model for the coordination of multiple complex systems

**Definition 2.** *Multiple complex systems*. *A complex system consists of many (n* ≥ 3) *subsystems*.

**Definition 3.** *Coordination degree of the complex system*. *This is an index describing the overall state of the system coordination*. *Assuming the state space and random state of the complex system are X and x*, *respectively*, *where x* ∈ *X*, *then the coordination is actually a scalar function which describes the system state with the corresponding state x*, *as*:

$$CD_{x}=f\left(x\right)$$ (3)

where *CD*_*x*_ s the coordination degree of the complex system under state *x*, and *f* is a real function defined by *X*.

**Fig 3:**
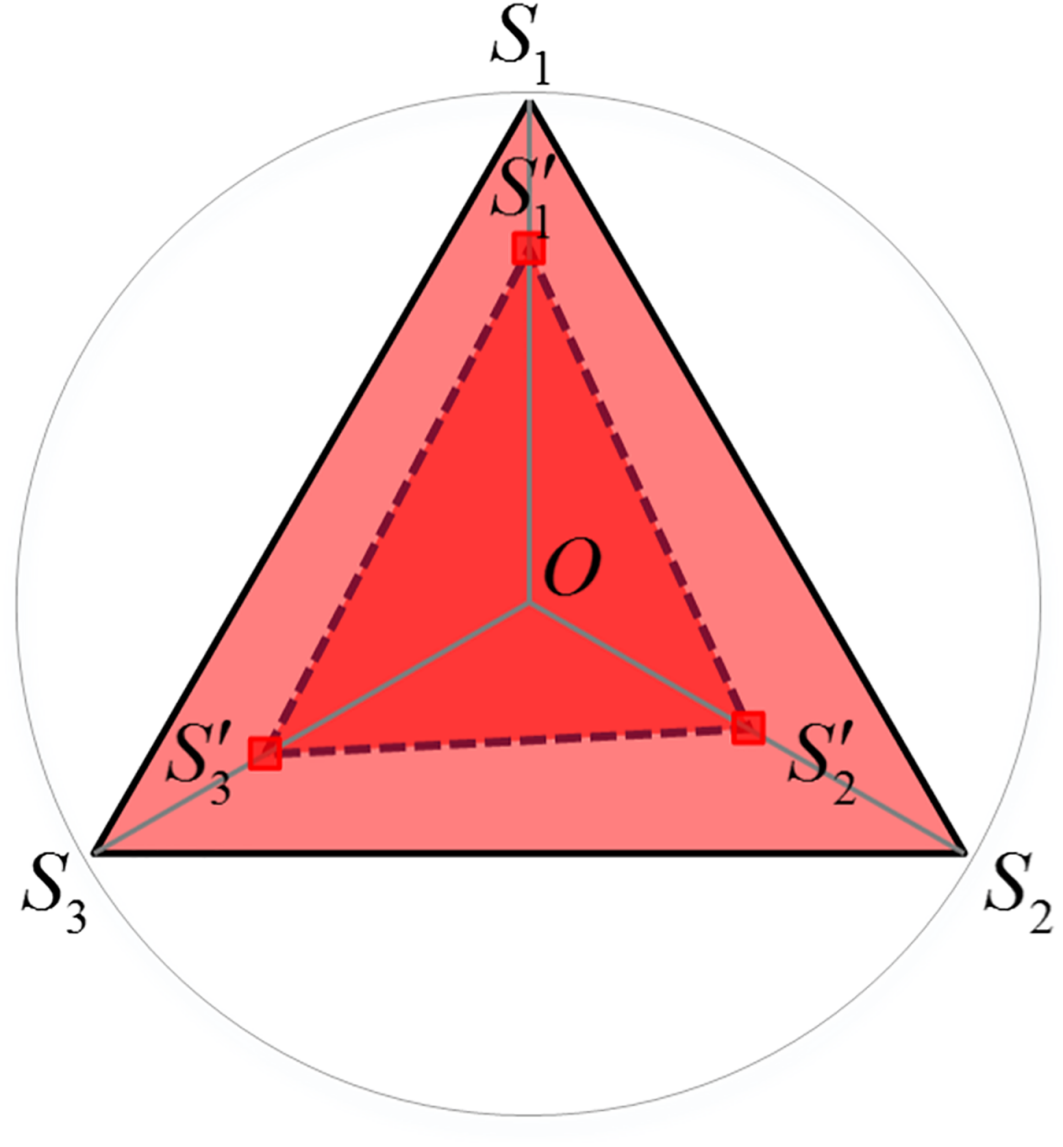
Calculation of the coordination degree of multiple complex systems.

During our investigation, the complex system is an organic whole. The integrated function is not equal to a simple sum of the functions of the different parts, but equal to that of every subsystem function adding the structural functions obtained from subsystem interactions. A ternary complex system serves as an example to introduce the calculation method for coordination degree of the complex system (Fig 3).

1. Draw a circle *O* with a radius of 1 and a equilateral triangle by setting subsystems *S*_1_, *S*_2_ and *S*_3_ as its vertices.
2. Set the central point of the circle *O* as the starting point, and, based on the development level of subsystems, measure a corresponding length on lines *OS*_1_, *OS*_2_ and *OS*_3_, respectively. Draw a new triangle $S_{1}^{\prime }S_{2}^{\prime }S_{3}^{\prime }$ with the intersections $S_{1}^{\prime }$, $S_{2}^{\prime }$ and $S_{3}^{\prime }$ as the vertices.
3. The computing equation for the coordination degree of the ternary complex system is:

$$CD=\sqrt{S_{S_{1}^{\prime }S_{2}^{\prime }S_{3}^{\prime }}/S_{S_{1}S_{2}S_{3}}}$$ (4)

where $S_{S_{1}S_{2}S_{3}}$ and $S_{S_{1}^{\prime }S_{2}^{\prime }S_{3}^{\prime }}$ are the areas of triangles *S*_1_*S*_2_*S*_3_ and $S_{1}^{\prime }S_{2}^{\prime }S_{3}^{\prime }$, respectively; *CD*_1_ ∈ [0,1]. When $S_{S_{1}^{\prime }S_{2}^{\prime }S_{3}^{\prime }}=S_{S_{1}S_{2}S_{3}}$, the coordination degrees of all subsystems are equal to 1 and the subsystem coordination degree *CD* = 1. All the subsystems are in an ideal state of coordination.

#### The coordination evaluation model of CSGC

**Fig 4:**
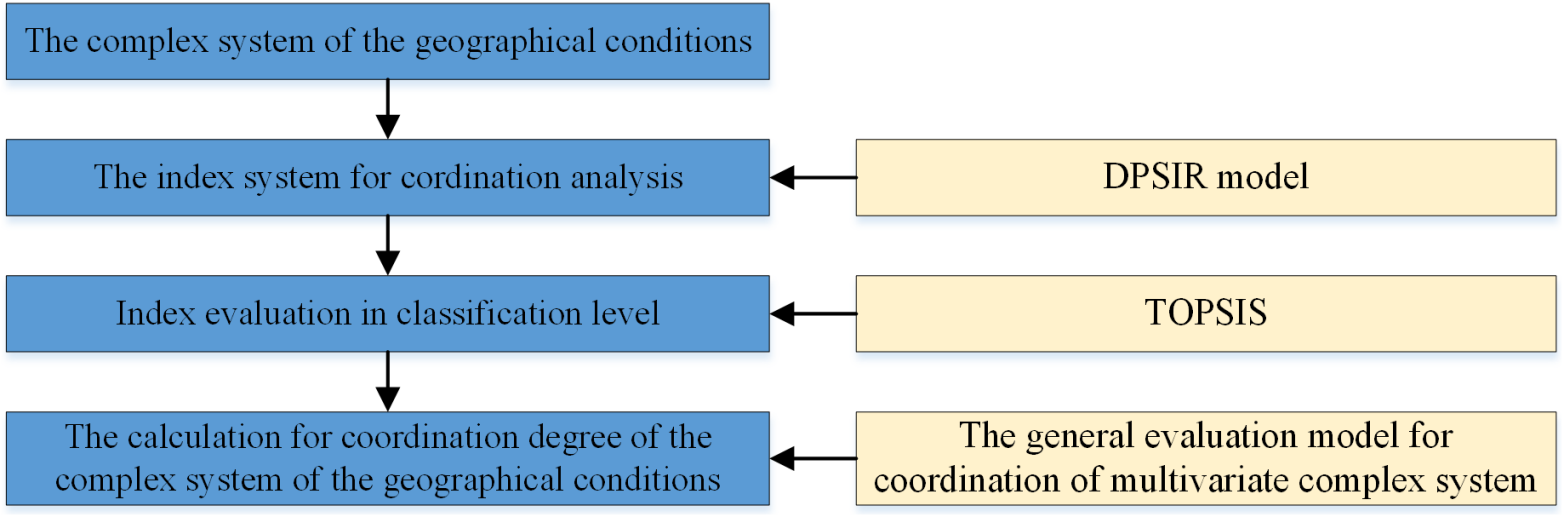
The coordination evaluation model of the CSGC.

The coordination evaluation of CSGC is transformed into the coordination analysis of the driving force, pressure, state, influence and response index in the DPSIR model (Fig 4). First, we construct the DPSIR index system and then evaluate the driving force, pressure, state, influence and response index. Then, we measure the coordination degree of CSGC using the general evaluation model for coordination of multiple complex systems according to the index evaluation results.

#### Classification index evaluation

The data matrix of classification index *i*, composed of *n* samples and *m*_*i*_ indices (indices selected from the classification index *i*), is developed and expressed as $X_{i}={\left(x_{jk}\right)}_{n\times m_{i}}$, where *x*_*jk*_ is the *k*^*th*^ index value of the *j*^*th*^ sample of classification index *i*. Before performing classification index evaluation, therefore, the impact of dimension and magnitude differences of the index data must be eliminated by separately normalizing the positive, negative and moderate indexes to achieve the communalities of evaluation indexes.

$$\begin{array}{l} \text{Positive index}\;:\;x_{jk}^{\prime }=\frac{x_{jk}-\min\;x_{jk}}{\max\;x_{jk}-\min\;x_{jk}}\\ \text{Negative index}\;:\;x_{jk}^{\prime }=\frac{\max\;x_{jk}-x_{jk}}{\max\;x_{jk}-\min\;x_{jk}}\\ \text{Appropriate index}\;:\;x_{jk}^{\prime }=1-\frac{\left|x_{jk}-x_{0k}\right|}{\max\left|x_{jk}-x_{0k}\right|} \end{array}$$ (5)

where indexes that have positive effectiveness are called positive indices, and vice versa, some indexes may go through a conversion of positive and negative effectiveness, especially with the variation of indexes and the development of the complex system. The effectiveness of selected indexes is positive when the index value approaches a specific value, and negative when they approach other specific values. These indexes are called appropriate indices. The max *x*_*jk*_, min *x*_*jk*_ and *x*_0*k*_ are the maximum, minimum and appropriate values of the *k*^*th*^ index value of the classification index *i*, respectively.

**Definition 4.** *Classification index ideal solution. All index values of a classification index are the maximum values of a normalized data sample and expressed by the vector:* $X_{i}^{+}=\left(\max\;x_{j1},\max\;x_{j2},\cdots ,\max\;x_{jm_{i}}\right)$.

**Definition 5.** *Classification index negative ideal solution. All index values of a classification index are the minimum values of a normalized data sample and expressed by the vector:* $X_{i}^{-}=\left(\min\;x_{j1},\min\;x_{j2},\cdots ,\min x_{jm_{i}}\right)$.

The nature of the index evaluation in the classification hierarchy is the degree of closeness to the ideal solution and distance from the negative ideal solution. This degree is generally measured by the distance or similarity. The calculation steps of the index evaluation in the classification hierarchy using TOPSIS are:

1 Calculate the distances from the classification index *i* to its ideal and negative ideal solution, respectively. Adopt the Euclidean distances, i.e., $d_{i}^{+}$ and $d_{i}^{-}$ between classification index *i* vector for each sample and use vectors $X_{i}^{+}$ and $X_{i}^{-}$ to express them, respectively (Eq 6). Thus;

$$d_{i}^{+}=\sqrt{\sum_{j=1}^{m_{i}}{\left(x_{jk}^{\prime }-x_{jk}^{\prime +}\right)}^{2}};d_{i}^{-}=\sqrt{\sum_{j=1}^{m_{i}}{\left(x_{jk}^{\prime }-x_{jk}^{\prime -}\right)}^{2}}$$ (6)

2 Calculate the value *E*_*i*_ of classification index *i* considering the closeness between classification index *i* and the ideal solution and the separation between classification index *i* and the negative ideal solution (Eq 4). Then;

$$E_{i}=\frac{d_{i}^{-}}{d_{i}^{+}+d_{i}^{-}}$$ (7)

where *E*_*i*_ ∈ [0,1]. When the mean values of all indices within the classification index *i* are at their maximum, $d_{i}^{+}=0$ and $d_{i}^{-}=1$, thus, *E*_*i*_ = 1; the maximum value. This suggests the sample classification index *i* is in the ideal state. When the mean values of all indices within the classification index *i* are at their minimum, $d_{i}^{+}=1$ and $d_{i}^{-}=0$, thus, *E*_*i*_ = 0; the minimum value. This suggests the sample classification index *i* is in its lowest state.

#### The calculation for coordination degree of the complex system of the geographic conditions

**Fig 5:**
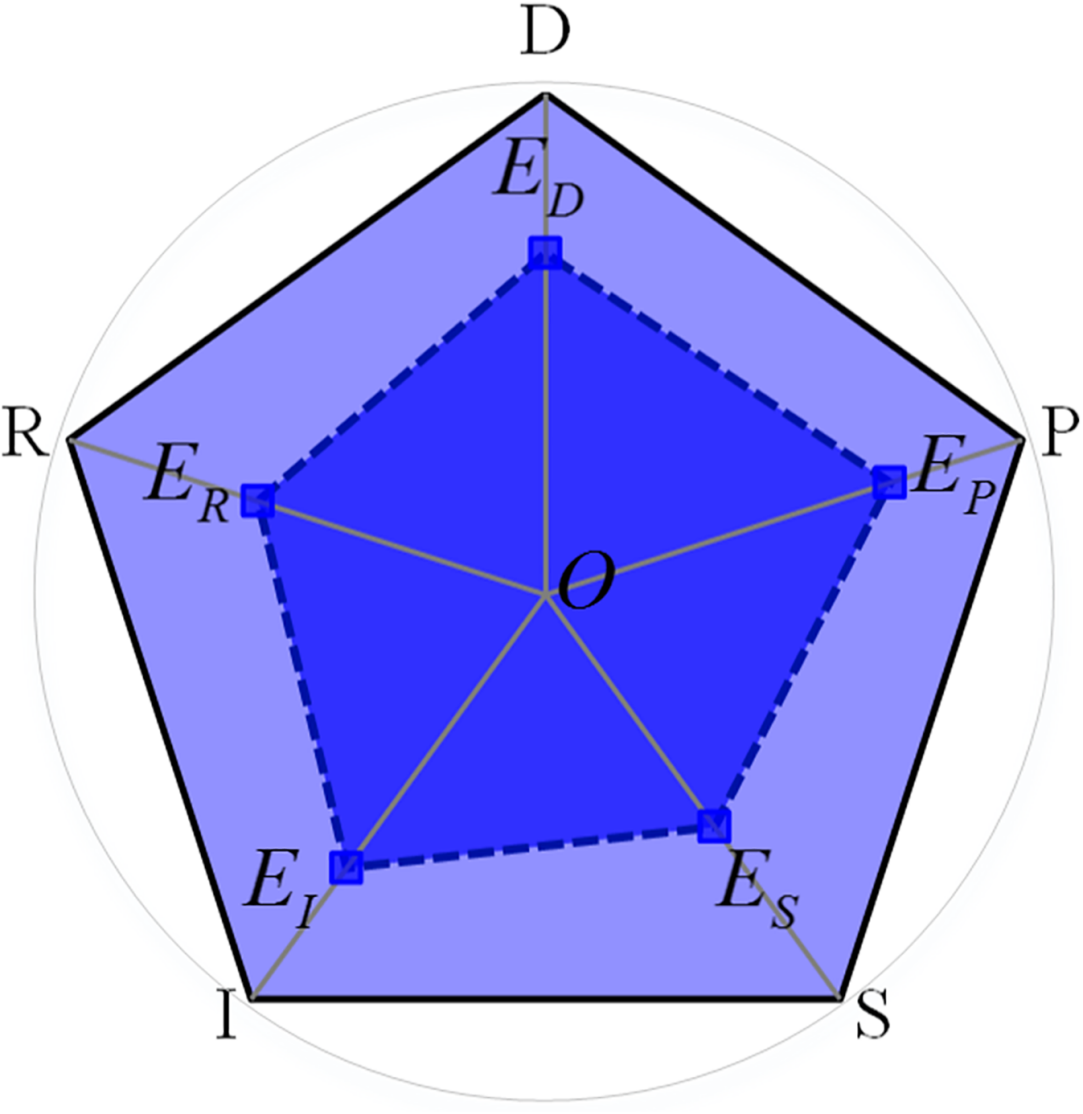
Calculation for the coordination degree of CSGC.

**Fig 6:**
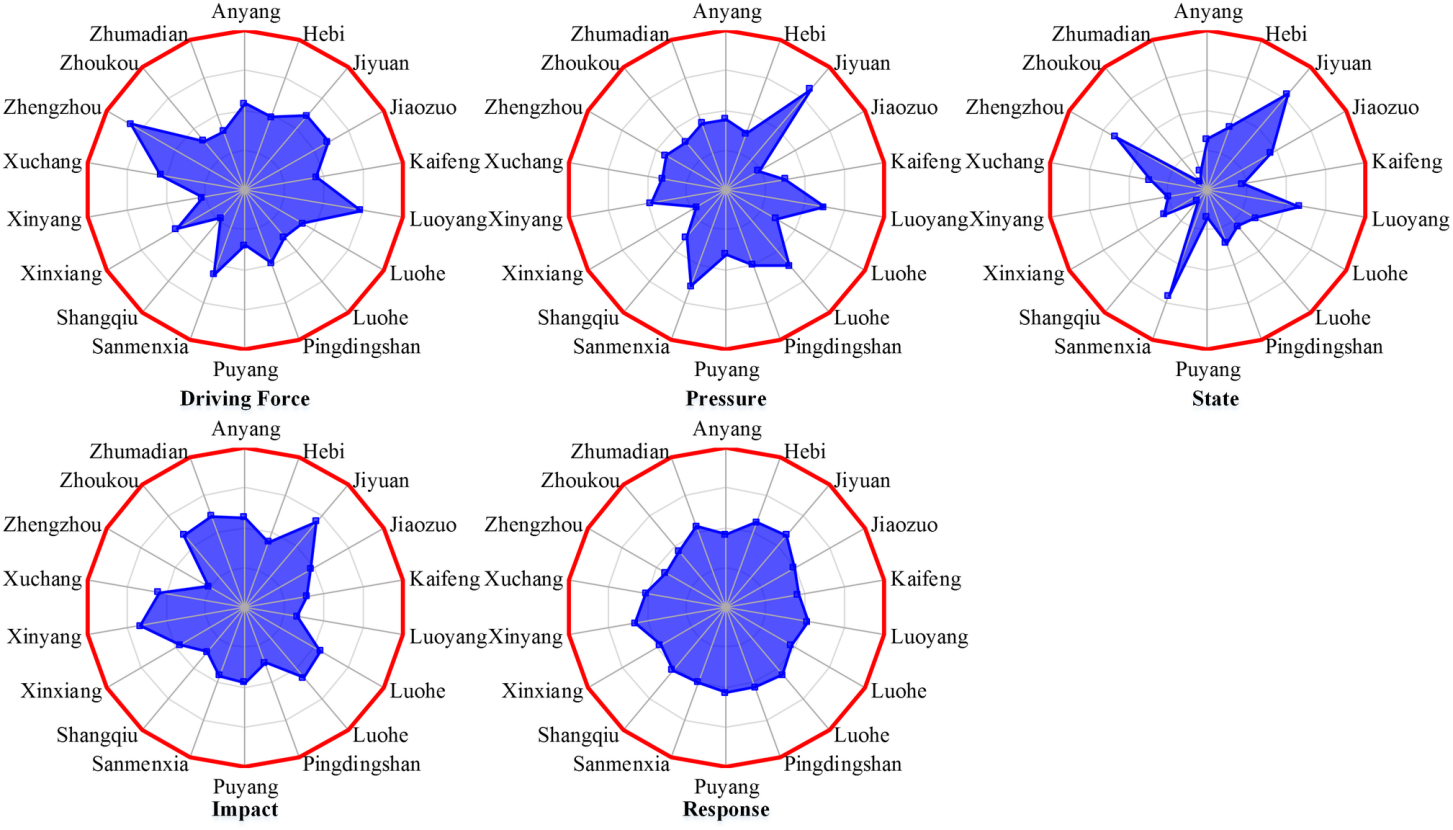
Regional evaluation for the classification index in the province of Henan.

According to the general evaluation model, the approach for the coordination calculation of the complex system of the geographic conditions is defined as (Fig 5):

$$CD_{GCCS}=\sqrt{S_{E_{D}E_{P}E_{S}E_{I}E_{R}}/S_{DPSIR}}$$ (8)

where $S_{E_{D}E_{P}E_{S}E_{I}E_{R}}$ and *S*_*DPSIR*_ are the areas of the pentagons *E*_*D*_*E*_*P*_*E*_*S*_*E*_*I*_*E*_*R*_ and *DPSIR*, respectively; *E*_*D*_, *E*_*P*_, *E*_*S*_, *E*_*I*_ and *E*_*R*_ in Fig 6 represent the evaluation values of the driving force, pressure, state, influence and response index, respectively. *OD* = *OP* = *OS* = *OI* = *OR* = 1.

## Results and discussion

**Fig 7:**
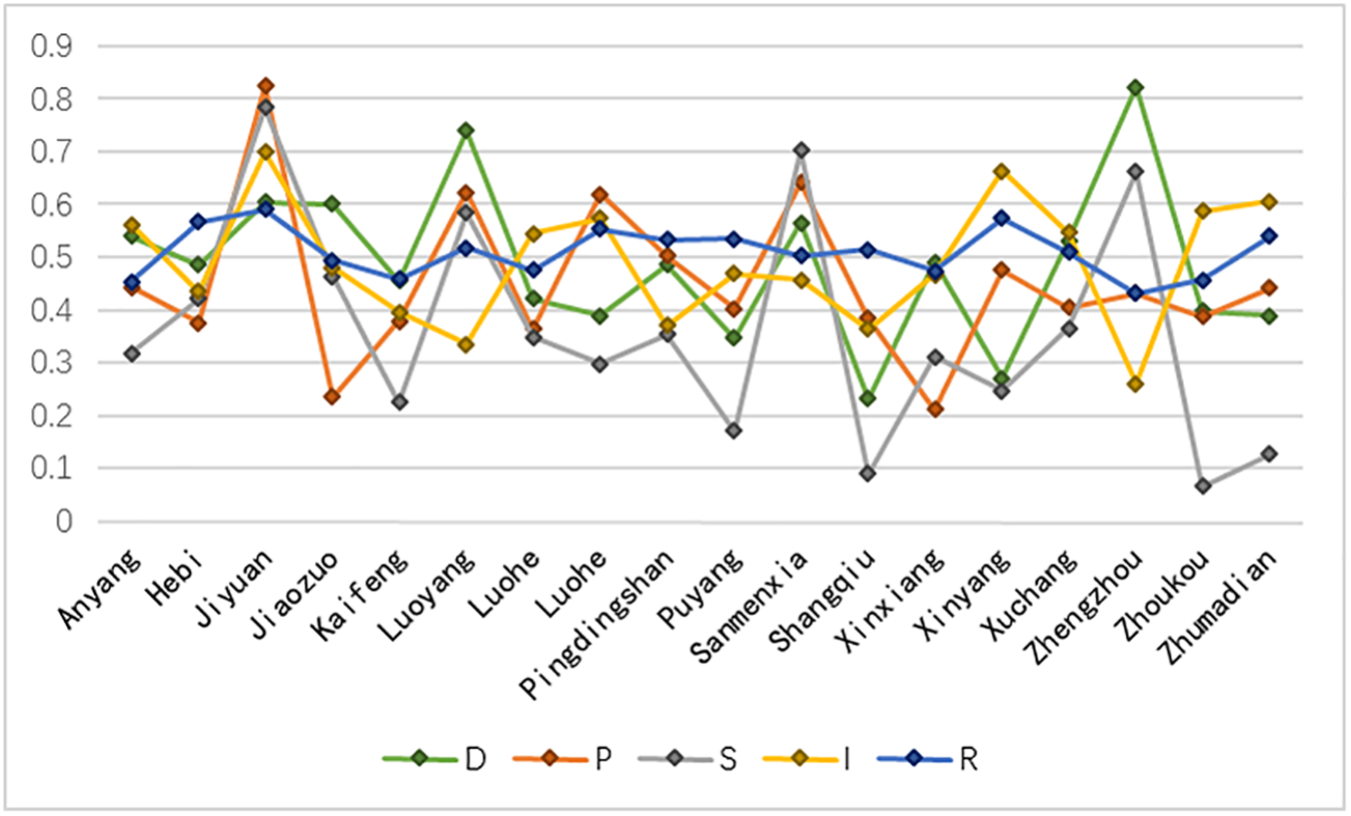
Classification index evaluation analysis of local cities in the Henan province.

The classification index evaluation and coordination degree for 18 administrative municipal divisions in the Henan province were determined and presented in Table 4. The classification is based on the index system and the designed evaluation model for coordination of the complex system of the geographic conditions. The leading five cities are Luoyang, Jiyuan, Jiaozuo, Luohe and Hebi, and the lowest five cities are Zhumadian, Zhoukou, Pingdingshan, Nanyang and Shangqiu. The regional evaluation radar map for the classification index (Fig 6) and the classification index evaluation analysis line chart of local cities (Fig 7) are constructed using the classification index and the region.

**Table 4:** Classification index evaluation and coordination degree for 18 administrative municipal divisions in the Henan province.

Administrative area	Classification index evaluation					*CD*_*GCCS*_
	D	P	S	I	R	
Anyang	0.539	0.4427	0.3173	0.5619	0.4535	0.4597
Hebi	0.4863	0.3758	0.4218	0.4343	0.567	0.4575
Jiyuan	0.6038	0.8245	0.7833	0.6998	0.5912	0.7018
Jiaozuo	0.6004	0.2356	0.4641	0.4805	0.4939	0.4489
Kaifeng	0.4547	0.3792	0.2244	0.3952	0.4585	0.3837
Luoyang	0.739	0.6228	0.584	0.3352	0.5181	0.5615
Luohe	0.4214	0.3632	0.3486	0.5452	0.4753	0.4311
Luohe	0.3884	0.6196	0.2975	0.5744	0.553	0.475
Pingdingshan	0.4849	0.5027	0.3537	0.3697	0.5324	0.4488
Puyang	0.3462	0.4033	0.1698	0.4689	0.5344	0.3803
Sanmenxia	0.5655	0.6411	0.7027	0.4573	0.5026	0.5742
Shangqiu	0.2319	0.3834	0.0898	0.3635	0.515	0.3041
Xinxiang	0.4913	0.2119	0.311	0.4674	0.4737	0.3923
Xinyang	0.2702	0.4748	0.2463	0.664	0.574	0.4348
Xuchang	0.5311	0.4047	0.3653	0.5457	0.5088	0.4712
Zhengzhou	0.539	0.4427	0.3173	0.5619	0.4535	0.4597
Zhoukou	0.4863	0.3758	0.4218	0.4343	0.567	0.4575
Zhumadian	0.6038	0.8245	0.7833	0.6998	0.5912	0.7018

Fig 6 reveals the response evaluation index in Henan Province is more balanced in terms of classification index. The pressure and state index evaluation are relatively unbalanced and vary greatly. Consistent measures taken in each city related to the driving force, pressure and impact of geographical conditions have different effects. Therefore, in the domain of sustainable development, every city should deploy special approaches to outstanding problems according to the local state, driving force and development pressure, and not pursue exhaustive measures or blindly follow and reference.

Fig 7 indicates large distinctions among the cities of Jiaozuo, Luoyang, Puyang, Shangqiu, Xinyang, Zhengzhou, Zhoukou and Zhumadian in the evaluation of the classification index value, while the variations were smaller for Anyang, Hebi, Jiyuan, Luohe, Pingdingshan, Sanmenxia and Xuchang. Combined with the coordination degree order of CSGC, We can find that geographical conditions coordination degree is low in cities with variation in classification index value (except Luoyang and Zhengzhou, the phenomenon shows that the extremum of coordination degree occurs mostly in the region of which the classification index value is large difference); on the contrary, geographical conditions coordination degree is high in cities with small differences in classification index value. The differences reflect the intensity of promoting or restricting effect between driving force, pressure, state, impact and response. If the difference is large, the intensity of interaction is large; if the difference is small, the intensity of interaction is small. The coordinated development of the complex system of geographical conditions needs to promote positive effects as much as possible, while inhibiting side effects.

**Fig 8:**
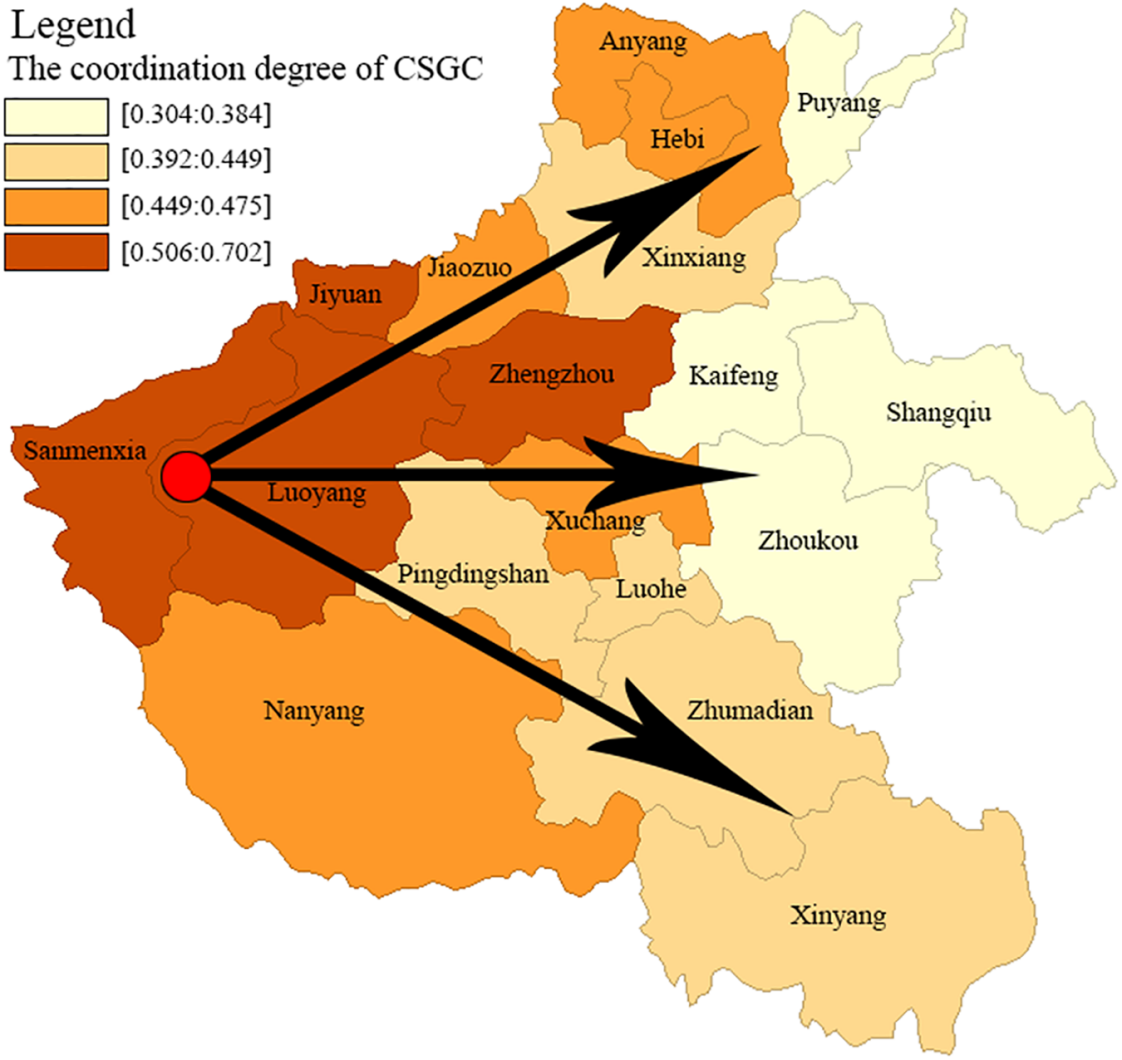
The hierarchical map of the coordination degree for CSGC in Henan province.

With the ArcGIS platform and the Natural Breaks Method, municipal regions in Henan province are classified into four categories according to the index value for D, P, S, I, R (Fig 8). Coordination levels are categorized as high, moderate, low, and very low. The evaluation results are presented in Table 5. From the results of Fig 8, we conclude the coordinated development of CSCG in the Henan province is at the stage of the point axis and a radial pattern initiated form Sanmenxia and Luoyang diverging to the northeast, east and southeast. The coordinated development center of CSCG focuses on a few good. The spatial correlation of geographical conditions elements and the classification index generate an assimilation effect with Sanmenxia, Luoyang, Zhengzhou and Jiyuan as the hot spots. Three geographical conditions coordinated development axis are gradually formed: ①Sanmenxia → Luoyang → Jiyuan → Jiaozuo → Xinxiang → Hebi → Anyang → Puyang (the coordination developed axis); ② Sanmenxia → Luoyang → Zhengzhou → Xuchang → Kaifeng → Zhoukou → Shangqiu (the coordination developing axis); ③ Sanmenxia → Luoyang → Nanyang → Pingdingshan → Luohe → Zhumadian → Xinyang (the coordination preliminary developing axis).

**Table 5:** The hierarchical description of the coordination degree for geographical conditions in Henan province.

Hierarchy		City
High coordination	0.5058–0.7018	Jiyuan, Luoyang, Sanmenxia
Moderate coordination	0.4349–0.5057	Anyang, Hebi, Jiaozuo, Nanyang, Pingdingshan, Xuchang, Zhenzhou
Low coordination	0.3838–0.4348	Luohe, Xinxiang, Xinyang, Zhumadian
Very low coordination	0.3041–0.3837	Puyang, Kaifeng, Shangqiu, Zhoukou

**Fig 9:**
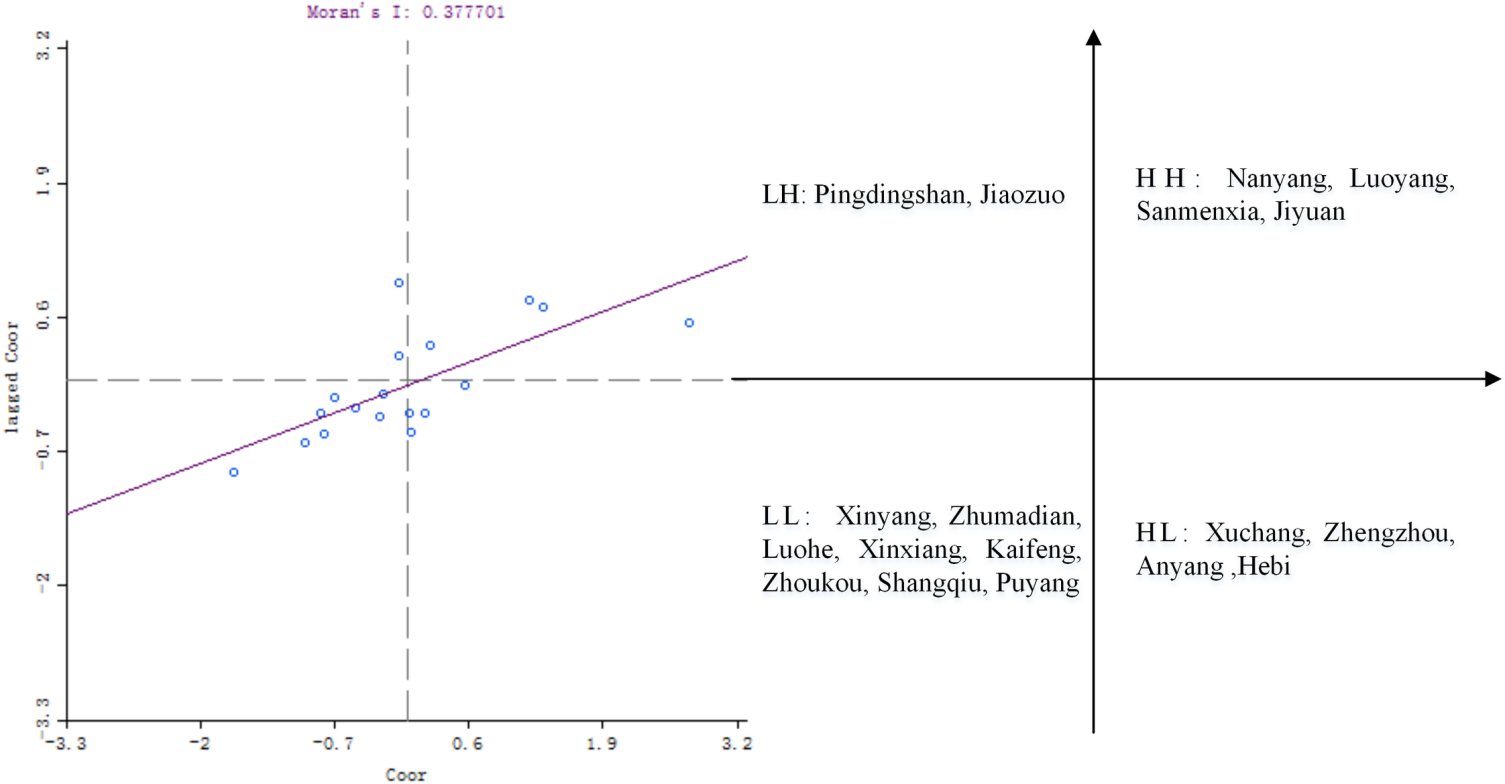
Moran scatter plot for the coordination degree of CSGC in the Henan province.

**Fig 10:**
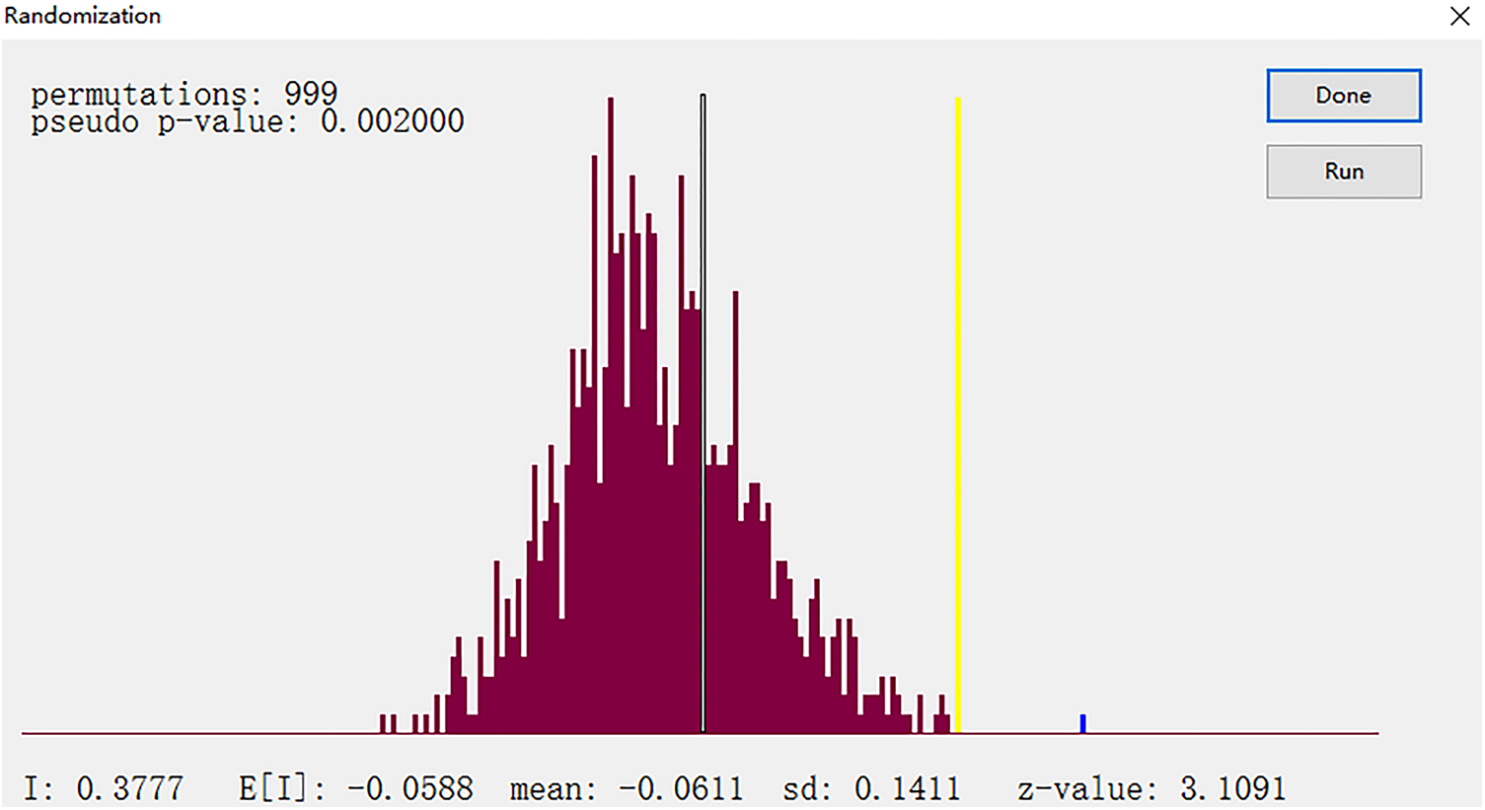
Significance level test of spatial correlation.

**Fig 11:**
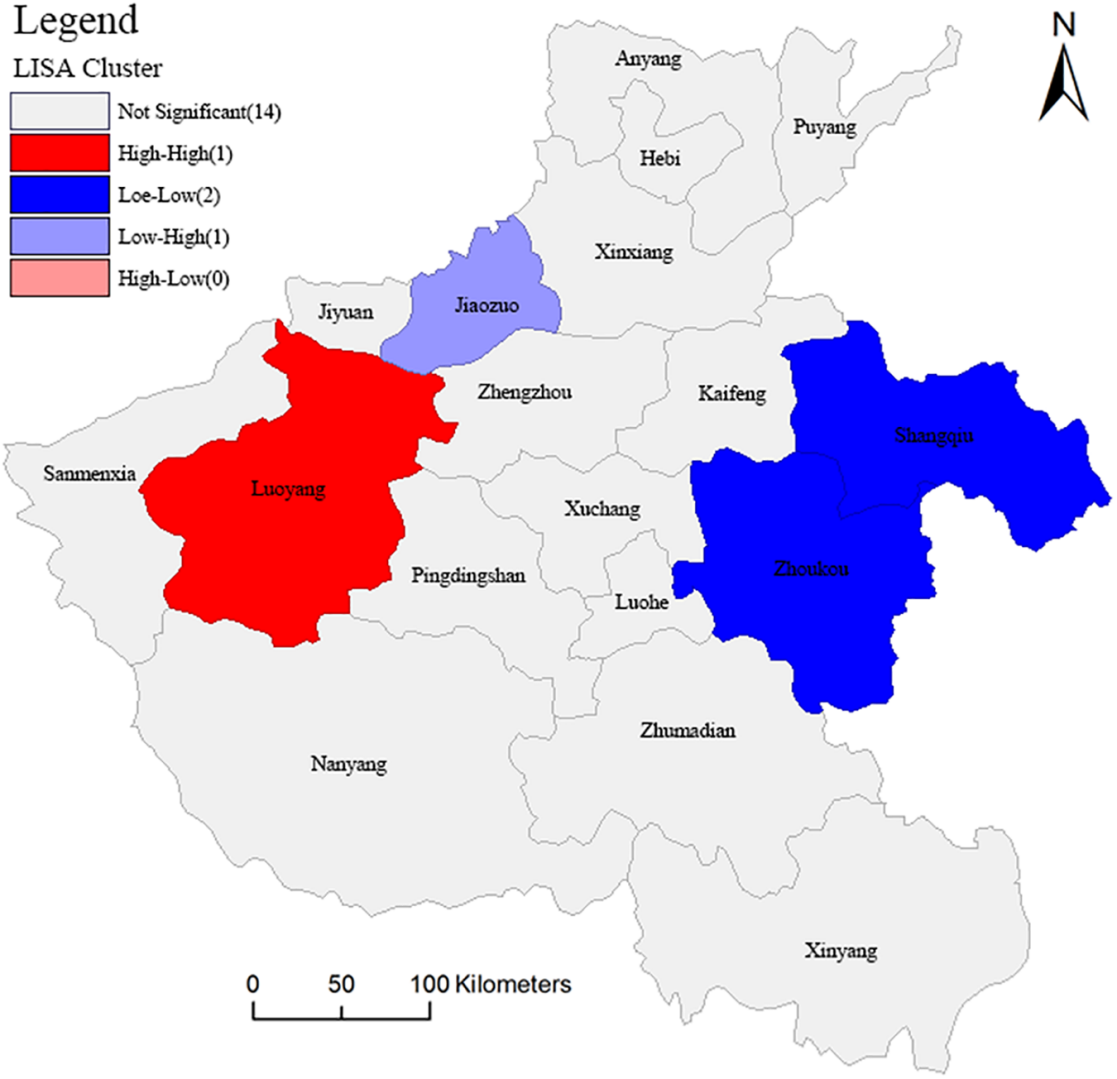
LISA cluster map for the coordination degree of CSGC in the Henan province.

The spatial correlation analysis in the Henan province is based on the calculated coordination degree of CSGC in order to reflect the spatial distribution, spatial impact, and spatial interactions of the coordination CSGC, and reveal objectively and accurately its spatial distribution rules and evolution trends. The global spatial autocorrelation can provide representative trends throughout the study area, assuming homogeneity of space conditions. However, the study area is often inhomogeneous and the local spatial autocorrelation can determine the heterogeneity [38]. Therefore, we used the GeoDa software to perform a global Moran's I and LISA (Local indicators of Spatial association) spatial relation analysis for the coordination of CSGC in the Henan Province, which sets permutation = 999. The Moran scatter plot, significance level test of spatial correlation and Lisa cluster map are shown in Figs 9, 10 and 11, respectively. This paper mainly analyzes the spatial autocorrelation of the coordination of CSGC between cities that the geographic location is adjacency, and adjacency between cities in Henan province are characterized by the edge of the adjacency, so rook is selected to apply in locational proximity matrix. The LISA is divided into Local Moran'I and Geary 'C indexes, and we used the former. The equations for the global Moran 's' I and Local Moran'I are:

$$I=\frac{n\sum_{i=1}^{n}\sum_{j=1}^{n}w_{ij}\left(X_{i}-\bar{X}\right)\left(X_{j}-\bar{X}\right)}{\sum_{i=1}^{n}\sum_{j=1}^{n}w_{ij}\sum_{i=1}^{n}{\left(X_{i}-\bar{X}\right)}^{2}}$$ (9)

$$I_{i}=\frac{\left(X_{i}-\bar{X}\right)}{S^{2}}\sum_{j}w_{ij}\left(X_{j}-\bar{X}\right)$$ (10)

where $\bar{X}=\frac{1}{n}\sum_{i=1}^{n}X_{i}$, *w* is the weight matrix.

The Moran plot and the significance level test indicate: ① The Moran'I value is 0.377701 and Z value is 3.1091, > 0, indicating the coordination of CSGC in the Henan province showed a global positive correlation; ② The coordination showing high-high spatial autocorrelations characteristics include the cities of: Nanyang, Luoyang, Sanmenxia and Jiyuan, a low-high autocorrelation characteristics for the cities of: Pingdingshan and Jiaozuo, a low-low spatial autocorrelation characteristics for the cities of: Xinyang, Zhumadian, Luohe, Xinxiang, Kaifeng, Zhoukou, Shangqiu and Puyang, and a high-low spatial autocorrelation characteristics for the cities of: Xuchang, Zhengzhou, Anyang and Hebi.

The value of Moran'I and Z have determined the existence of the relevance. Moran scatterplot qualitatively describes the correlation between cities those are adjacent, and the 18 cities, respectively, fall into the corresponding quadrant. But Moran scatterplot can't reveal the difference of the spatial autocorrelation degree, LISA cluster map can further explain the specific spatial location and significance of the cluster. In this paper, the LISA cluster map (Fig 11) is produced at a significant level of *P* ≤ 0.5 and clearly shows the local spatial autocorrelation regions. Fig 11 reveals: ① The Luoyang area and its surroundings regions displayed significant high-high spatial autocorrelation characteristics; ② The Shangqiu and Zhoukou regions and surrounding areas showed significant low-low spatial autocorrelation characteristics; ③ The Jiaozuo region and surrounding areas defined significant low-high spatial autocorrelation characteristics.

According to the coordinated development situation of geographical conditions and the results of spatial autocorrelation analysis, and then determine the specific improvement direction based on the classification index evaluation value, and formulate appropriate measures. Play the role of radiation in Luoyang that displayed significant high-high spatial autocorrelation characteristics, and use its driving force in order to promote the coordinated development of geographical conditions in surrounding cities. Focus on governance in Zhoukou and Shangqiu that showed significant low-low spatial autocorrelation characteristics, and suppress its impact on the surrounding cities to seek the driving force for development according to the status quo and the pressure.

## Conclusions

Previous research in geographical conditions statistical analysis evolved into gradually incorporating the harmonious development of complex systems into all aspects of creating a resource-saving, environment-friendly, ecologically-civilized and sustainably-developing society. This is important in studying geographical conditions. It is also an effective process in solving current problems faced by geographical conditions statistical analysis.

(1) Establishing a reasonable coordination analysis index system constitutes the basis of a scientific evaluation of geographical conditions. In this paper, a hierarchy structure of a coordination analysis index system was established using the DPSIR model. By analyzing the relationships between CSGC and impact factors, an optimized index classification program was determined both objectively and subjectively, thus the index system for the coordination analysis of geographical conditions was established. The indexes were selected on the goal of the coordination analysis of CSGC, and their connotations and indicative functions are clear. The hierarchical structure of the index system, consisting of target, classification, and index hierarchies, is explicit. There are no indexes isolated outside the system. The indexes were also selected from five components: resources, ecology, environment, economy, and society. This meets the requirements of statistical analysis of the census and the monitoring data for current geographic national conditions. It covers the four parts of the analysis and evaluation report for geographic national conditions, which includes resource distribution, ecological protection, regional economics, and social development. Therefore, our index system meets the requirements for comprehension and integrity. Each element of the hierarchy and factors (D, P, S, I, R) in the classification corresponds to different indexes and there is no overlap between the index sets for different factors. This meets the requirements for independence of indexes and non-overlapping factors. The driving force, pressure, state, impact, and response in the DPSIR model reveals the interactions of matter, energy, values, information transfer, and exchange.

(2) Analyzing the relations between the geographical conditions and the complex system enables to propose and quantitatively describe the CSGC concept. Coordination analysis methods have been introduced for geographical conditions and supported by the complex system theory. The complex system theory provided a theoretical guidance for researching geographical conditions. The degrees of coordination provided new ideas for measuring the relationships between the constituent elements of geographical conditions.

(3) The proposed evaluation method of coordination degree is general. Although the experiment is aiming to geographical conditions of Henan Province, for other provinces, we need to select and build the corresponding coordination evaluation index system according to the research content of the geographical conditions and the actual situation of the region.

(4) The proposed general coordination evaluation model of multivariate complex system in this paper, doesn’t take into account the effect of element order change on the final evaluation result of coordination, in the course of analysis and calculation using radar chart, and it should be improved in the future work.

(5) On the regionalization analysis to CSCG, only the results of the regionalization itself is analyzed, and without taking into account the comparative and correlation analysis with the other existing regionalization, such as geographical regionalization, economic regionalization, ecological regionalization, etc. These should be considered in the future work.

## Supporting information

S1 Dataset S1 Dataset used in the case study. This dataset includes the statistical yearbook of henan province in 2013(*.xls), and the administrative area of henan province(henan.shp).
(ZIP)
